# The R2R3 MYB transcription factor MdMYB30 modulates plant resistance against pathogens by regulating cuticular wax biosynthesis

**DOI:** 10.1186/s12870-019-1918-4

**Published:** 2019-08-19

**Authors:** Ya-Li Zhang, Chun-Ling Zhang, Gui-Luan Wang, Yong-Xu Wang, Chen-Hui Qi, Qiang Zhao, Chun-Xiang You, Yuan-Yuan Li, Yu-Jin Hao

**Affiliations:** National Key Laboratory of Crop Biology; Shandong Collaborative Innovation Center of Fruit & Vegetable Quality and Efficient Production; College of Horticulture Science and Engineering, Shandong Agricultural University, Tai-An, 271018 Shandong China

**Keywords:** Apple, Cuticular wax, *MdMYB30*, Pathogens resistance

## Abstract

**Background:**

The MYB transcription factor family is one of the largest transcriptional factor families in plants and plays a multifaceted role in plant growth and development. However, MYB transcription factors involved in pathogen resistance in apple remain poorly understood.

**Results:**

We identified a new MYB family member from apple, and named it *MdMYB30. MdMYB30* was localized to the nucleus, and was highly expressed in young apple leaves. Transcription of *MdMYB30* was induced by abiotic stressors, such as polyethylene glycol and abscisic acid. Scanning electron microscopy and gas chromatograph–mass spectrometry analyses demonstrated that ectopically expressing *MdMYB30* in Arabidopsis changed the wax content, the number of wax crystals, and the transcription of wax-related genes. *MdMYB30* bound to the *MdKCS1* promoter to activate its expression and regulate wax biosynthesis. *MdMYB30* also contributed to plant surface properties and increased resistance to the bacterial strain *Pst* DC3000. Furthermore, a virus-based transformation in apple fruits and transgenic apple calli demonstrated that *MdMYB30* increased resistance to *Botryosphaeria dothidea*. Our findings suggest that *MdMYB30* plays a vital role in the accumulation of cuticular wax and enhances disease resistance in apple.

**Conclusions:**

*MdMYB30* bound to the *MdKCS1* gene promoter to activate its transcription and regulate cuticular wax content and composition, which influenced the surface properties and expression of pathogenesis-related genes to resistance against pathogens. MdMYB30 appears to be a crucial element in the formation of the plant cuticle and confers apple with a tolerance to pathogens.

**Electronic supplementary material:**

The online version of this article (10.1186/s12870-019-1918-4) contains supplementary material, which is available to authorized users.

## Background

Apple (*Malus × domestica*) is an important fruit crop that is commonly grown worldwide. The gloss and smoothness on apple are important traits that determine the market value of apple, as disease-free shiny fruits are more attractive to consumers. Cuticular wax is responsible for resistance to apple pathogens and gloss.

The plant cuticular structure is composed of two parts based on physical location: the cutin, which is close to the cell wall, and the epicuticular wax, which is exposed to the air. An epicuticular film of wax crystals covers the plant surface [1]. Cuticular wax plays crucial roles protecting against external environmental stress, such as acting as a transpiration barrier and functioning in interactions with pathogens. The basic constituents of plant cuticular wax are very long chain fatty acids (VLCFAs) and their derivatives. The cuticular synthetic pathway can be divided into three reactions: (1) de novo synthesis of C16 and C18 fatty acids; (2) extension of VLCFAs: the C16 and C18 fatty acids produced during the first stage extend on the endoplasmic reticulum to form C20–C36 VLCFAs; and (3) synthesis of derivatives of VLCFA, such as aldehydes, alcohols, alkanes, ketones, and esters [2].

The function of epicuticular wax as a transpiration barrier has been widely investigated. In maize, removing the wax layer on the surface of wild maize promotes the formation of adherent cells of rust on the leaf surface [3]. In addition, the epidermal wax on the surface of the leaf can be used to defend against natural enemies [4]. Some lipids may act as signaling substances during cell apoptosis and defend against external invasion [5].

MYB TFs are common among all eukaryotes; however, this protein family is particularly large in higher plants. The MYB protein family in animals contains three MYB conserved domain repeats (R1, R2, and R3), whereas most plant MYB TFs belong to the R2R3 type, which are associated with growth regulatory processes [6]. MYB proteins are reportedly related to a series of functions regulating secondary metabolism, such as epidermal wax [5], morphogenesis [7], and the abiotic and biotic stress responses [8, 9]. The functions of the MYB family members have been widely studied. Overexpressing *MYB94* increases total wax load approximately two-fold in Arabidopsis leaves, as *MYB94* binds to the promoters of wax-related genes to enhance their expression [10]. Programmed cell death is a hypersensitive response that is closely related to plant disease resistance. *AtMYB30* is an active modulator of cell death in both avirulent and virulent pathogen attack responses [11]. MYB30, MYB55, and MYB110 are three MYB proteins that are transcriptionally induced by microbe-associated molecular pattern (MAMP) treatment, which enhance resistance to fungal and bacterial pathogens and have important roles in rice plant immunity [12]. Furthermore, *AtMYB30* shows great response to reactive oxygen species (ROS) and inhibits root cell elongation. This process involves multiple genes associated with VLCFA transport; thus, providing a molecular link between ROS - root cell - wax biosynthesis - plant immune responses [13].

The cuticle of fruit crops supplies morphological support for the integrity of the entire fruit and also affects growth and maturation [14]. The composition and content of cuticular wax may lead to differences in quality characteristics, such as post-harvest resistance to water loss, pathogen infection, and cracked fruit [15–17]. A large number of studies on fruit wax have concentrated on wax morphology and biosynthesis, whereas little is known about the molecular pathways regulating wax biosynthesis and their roles in apple. Here, we identified an apple R2R3 MYB TF named *MdMYB30*. Furthermore, ectopically expressing *MdMYB30* improved disease resistance by changing the composition and surface properties of the wax. These results demonstrate that *MdMYB30* positively regulates wax biosynthesis and acts as a pathogen resistance mechanism.

## Results

### Identification of the *MYB30* gene in apple

**Fig. 1:**
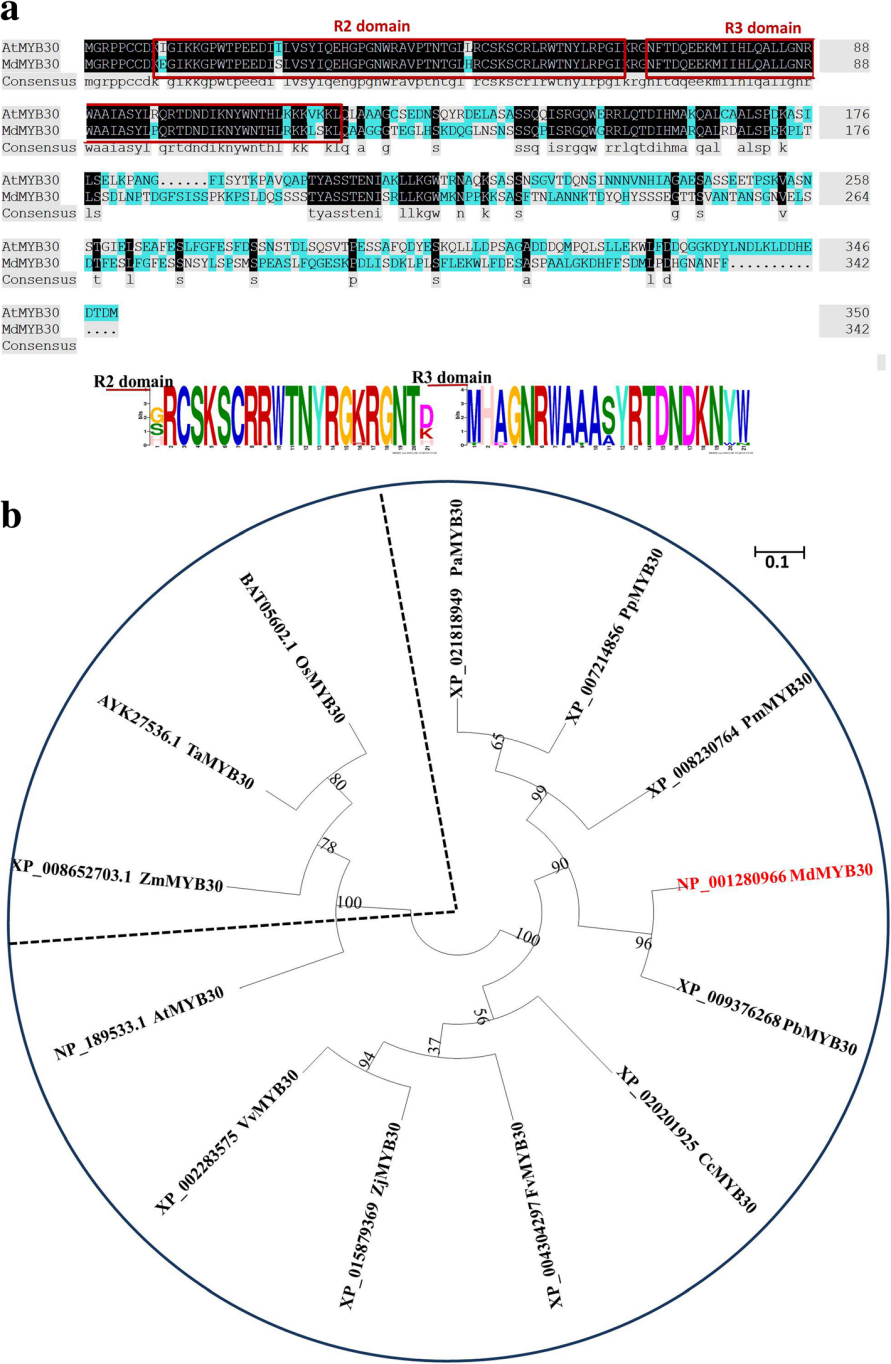
Sequence alignment and phylogenetic analysis of MdMYB30. **a** Alignment of the MdMYB30 and AtMYB30 amino acid sequences. The locations of two conserved motifs are labeled with red lines. The conserved R2 and R3 domains required for DNA binding of MdMYB30 and AtMYB30 proteins. Conservation of residues across the MdMYB30 and AtMYB30 proteins is indicated by the height of each letter. Bit scores indicate information for each conserved motif in the sequence (To interpret the colors in this legend, please refer to the web version of this article). **b** Phylogenetic relationship analysis of the plant MYB30 proteins. Phylogenetic analysis of MdMYB30 and 12 other plants MYB30 protein sequences obtained from the NCBI database. MdMYB30 is denoted in red font, and the scale bar indicates the branch length. MdMYB30: NP_001280966 *Malus domestica*; PbMYB30: XP_009376268 Pyrus bretschneideri; PmMYB30: XP_008230764 *Prunus mume*; PaMYB30: XP_021818949 *Prunus avium*; PpMYB30: XP_007214856 *Prunus persica*; FvMYB30: XP_004304297 *Fragaria vesca*; ZjMYB30: XP_015879369 Ziziphus jujube; VvMYB30: XP_002283575 Vitis vinifera; CcMYB30: XP_020201925 *Cajanus cajan*; TaMYB30: AYK27536.1 *Triticum aestivum*; OsMYB30: BAT05602.1 *Oryza sativa*; ZmMYB30 XP_008652703.1 *Zea mays*; AtMYB30:NP_189533.1 *Arabidopsis thaliana*

To separate the *MdMYB30* gene, the *AtMYB30* gene was used as bait to search sequences in apple based on homologous retrieval. We chose a gene with the highest homology to AtMYB30, and named it MdMYB30 (MDP0000149102). The *MdMYB30* cDNA contained two noncoding regions (Additional file 1: Figure S1), and the *MdMYB30* ORF encoded a 342 amino acid polypeptide as predicted by DNAMAN 6.0 software [18]. We analyzed the amino acid sequences of MdMYB30 and AtMYB30. Figure 1a shows that the MdMYB30 and AtMYB30 protein sequences were highly similar and contained conserved R2R3 domains.

A phylogenetic tree was constructed to investigate the evolutionary relationship of MYB30s among species (Fig. 1b; Additional file 1: File S1). The results indicated that apple MdMYB30 exhibited the closest evolutionary relationship with pear PbMYB30 (XP_009376268), because they were in the same clade. MdMYB30 had the most distant relationship with maize ZmMYB30 (XP_008652703.1), wheat TaMYB30 (AYK27536.1), and rice OsMYB30 (BAT05602.1), as all three are monocotyledonous plants. The phylogenetic tree indicated a clear boundary between dicotyledons and monocotyledons.

### MdMYB30 is localized in the nucleus and is induced by abiotic stress

**Fig. 2:**
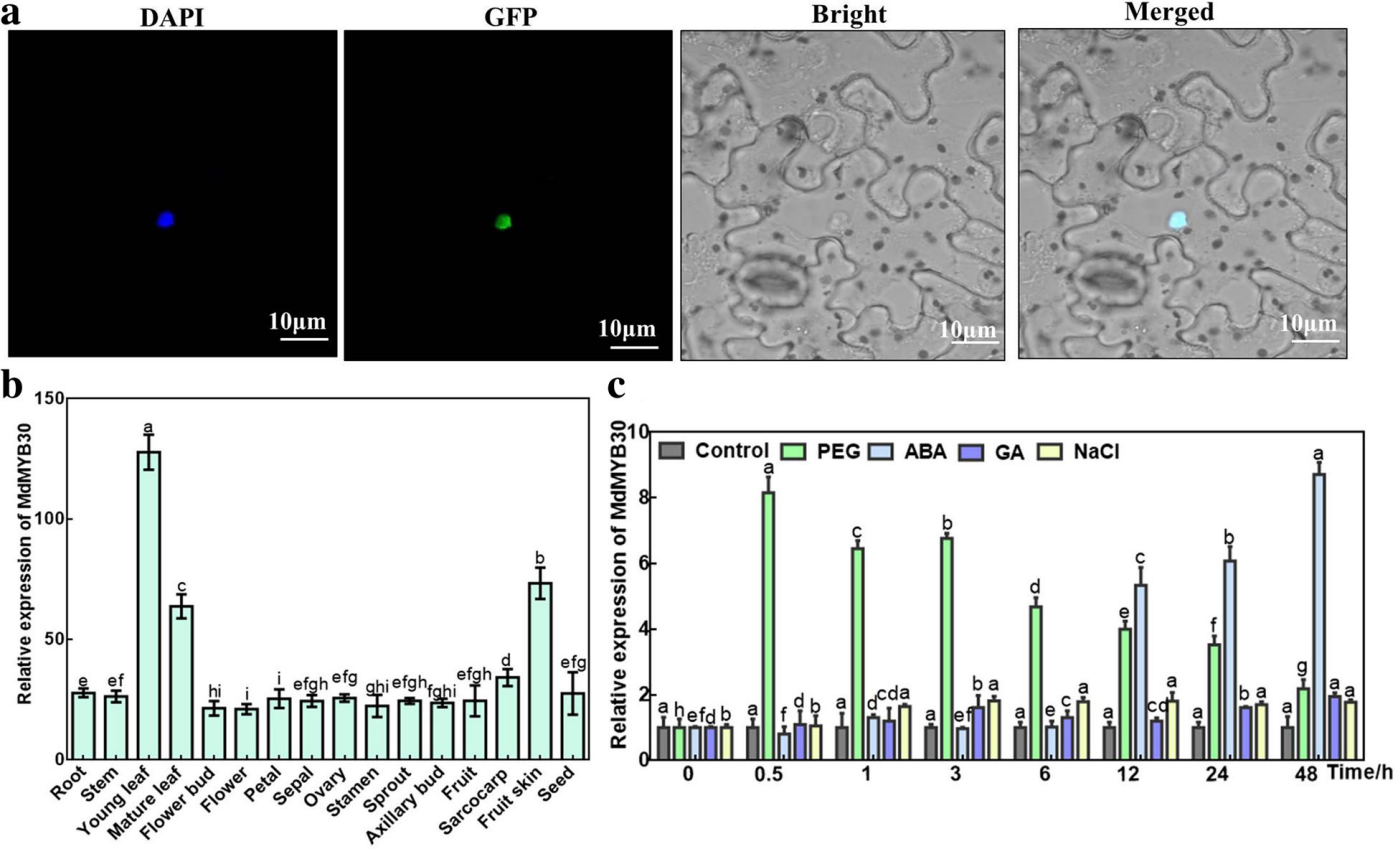
Subcellular localization and expression patterns of *MdMYB30.* **a** Subcellular localization of the MdMYB30 protein in *Nicotiana benthamiana*. Transient expression of the 35S:MdMYB30-GFP fusion construct in *N. benthamiana*. Green fluorescence was observed 3 days after transient infection using a confocal microscope. 4′,6-diamidino-2-phenylindole (DAPI) dyes specifically stain the nucleus, while the fusion protein green fluorescence completely overlaps with the nucleus (scale bar: 10 μm). **b** Expression profile of *MdMYB30* in various apple tissues (Root, stem, young and mature leaves, floral bud, flower, petal, sepal, ovary, stamen, sprout, axillary bud, fruit, sarcocarp, fruit skin, and seed). Apple actin expression was used as the control. **c** Expression level of *MdMYB30* in response to PEG, ABA, GA, and NaCl. Error bars represent standard deviations (SD; *n* = 3). Data are mean ± SD of three independent replicates. Different lowercase letters indicate significant differences at *P* < 0.05

The localization of a protein is very important for its function. Figure 2a indicates that the green fluorescent signal and the blue fluorescent signal merged in the nucleus, demonstrating that MdMYB30 localized to the nucleus. The *MdMYB30* transcription analysis in various apple tissues showed that although it was constitutively expressed in all examined tissues, the expression levels were different, indicating the specific function of *MdMYB30* in its highly expressed tissues (Fig. 2b).

To begin to investigate the *MdMYB30* stress response, *cis-*elements in the *MdMYB30* promoter were generated by applying PlantCARE software [19]. A number of regulatory sequences in the *MdMYB30* promoter were identified, including elements involved in heat, low-temperature, and drought stressors, elements involved in plants, such as SA, ABA, and GA, and several light-responsive elements (Table 1), suggesting that *MdMYB30* is closely related to plant response to various abiotic stresses.

**Table 1:** Promoter cis-acting element analysis of *MdMYB30*

Regulatory sequence	sequence	Function of site	Location
ABRE	GCCGCGTGGC	cis-acting element involved in the abscisic acid responsiveness	− 119
ACE	AAAACGTTTA	cis-acting element involved in the abscisic acid responsiveness	+ 259
ARE	AAAACGTTTA	cis-acting regulatory element essential for the anaerobic induction	+ 373
G-box	CACGAC	cis-acting regulatory element involved in light responsiveness	+ 486
GARE-motif	TCTGTTG	gibberellin-responsive element	+ 1357
GATA-motif	GATAGGG	part of a light responsive element	− 1390
HSE	AAAAAATTTC	cis-acting element involved in heat stress responsiveness	− 988
LAMP-element	CTTTATCA	part of a light responsive element	+ 792
LTR	CCGAAA	cis-acting element involved in low-temperature responsiveness	−376
MBS	CAACTG	MYB binding site involved in drought-inducibility	− 1173
P-box	CCTTTTG	gibberellin-responsive element	− 216
Sp1	CC(G/A)CCC	light responsive element	+ 1406
TC-rich repeats	ATTTTCTCCA	cis-acting element involved in defense and stress responsiveness	− 1305
TCA-element	GAGAAGAATA	cis-acting element involved in salicylic acid responsiveness	+ 268

Subsequently, we analyzed the expression of the *MdMYB30* gene treated with osmotic stress (PEG), ABA, GA, or NaCl, respectively (Fig. 2c). In vitro-propagated apple seedlings were used for this experiment. The *MdMYB30* transcript level increased rapidly peaking at 0.5-h, and then gradually declined in the PEG treatment. *MdMYB30* expression did not change during the early stage in response to ABA, but increased sharply after 12-h of treatment and reached its maximum at 48-h. No changes in the *MdMYB30* transcript levels were detected after the GA and NaCl treatments.

### *MdMYB30* functions in biotic and abiotic stress

**Fig. 3:**
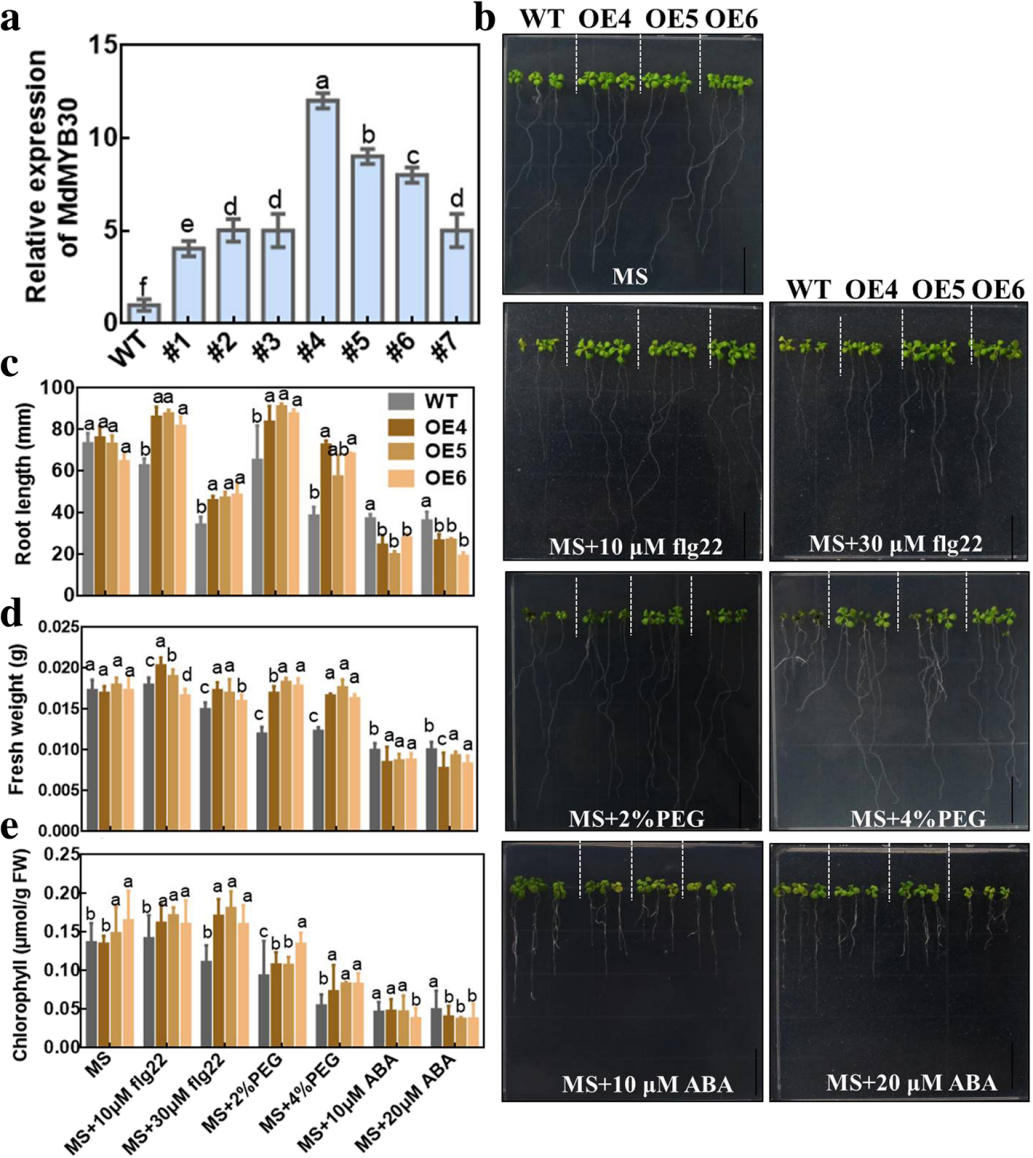
MdMYB30 participates in the biotic and abiotic stress response. **a** Identification of ectopically expressed *Arabidopsis thaliana* expression level of MdMYB30 by qRT-PCR. **b** Phenotype of wild-type and *MdMYB30* ectopically expressed Arabidopsis treated with or without 10 μM flg22, 30 μM flg22, 2% PEG, 4% PEG, 10 μM ABA, or 20 μM ABA (scale bar: 1 cm). **c-e** Root length **c**, fresh weight **d**, and chlorophyll content **e** in **b** Arabidopsis seedlings. Arabidopsis chlorophyll μmol/g FW (fresh weight = FW). Error bars represent standard deviation (SD; *n* = 3). Data are mean ± SD of three independent replicates. Different lowercase letters indicate significant differences at *P* < 0.05

We transformed *MdMYB30* into wild-type Arabidopsis to investigate the role of *MdMYB30* during stress. Six independent *MdMYB30* ectopically expressing (*MdMYB30* OE) Arabidopsis lines as determined by qPCR were obtained. *MdMYB30* OE4, OE5, and OE6 with higher *MdMYB30* transcription levels were selected for subsequent experiments (Fig. 3a). We monitored the flg22-induced immune response, PEG-induced osmotic stress, and ABA sensitivity of *MdMYB30* to ectopic expression in Arabidopsis. No clear differences were detected between WT and *MdMYB30* ectopic expression Arabidopsis in normal MS medium. *MdMYB30* ectopic expression roots were longer under the flg22 or PEG treatment than those in WT. However, the growth of roots in *MdMYB30* ectopic expression was significantly inhibited under ABA treatment, compared to the WT. The change in root elongation was more obvious with the increase of flg22, PEG, and ABA concentrations (Fig. 3b, c). Fresh weight and chlorophyll in the *MdMYB30* ectopic expression lines improved significantly after the flg22 and PEG treatments, whereas they decreased with the ABA treatment, compared to the WT, indicating that ectopic expression of the *MdMYB30* lines increased resistance to biotic stress, osmotic stress, and improved sensitivity to ABA (Fig. 3c-e). Therefore, these data suggest that *MdMYB30* participates in the response to biotic and abiotic stress.

### *MdMYB30* alters epicuticular wax load and composition

**Fig. 4:**
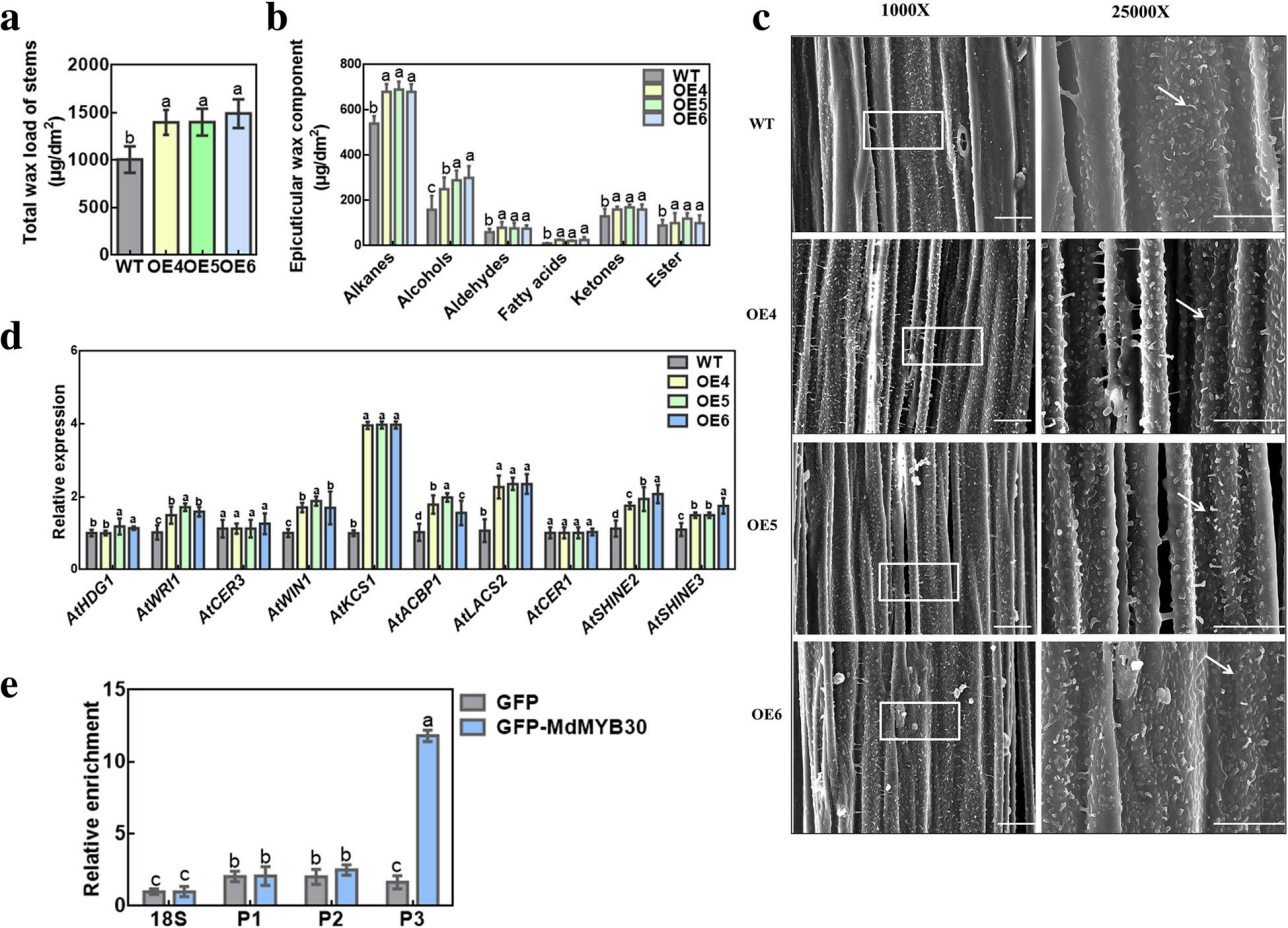
Changes in wax load, composition, cuticle wax crystal morphology, and expression of wax-related genes in WT and *MdMYB30* ectopic expression plants. **a** Total cuticular wax content of stems, calculated per unit area of 6-week-old Arabidopsis from WT and *MdMYB30* ectopic expression lines. **b** Cuticular wax composition of 6-week-old Arabidopsis stems for the WT and *MdMYB30* ectopic expression lines. **c** Wax crystal morphology of 6-week-old Arabidopsis stems from the WT and *MdMYB30* ectopic expression lines detected by scanning electron microscopy (scale bars: 10 μm). The surface of the *MdMYB30* ectopic expression lines were covered with wax crystals and wax deposition, whereas the wild-type surface showed only a small amount of wax deposition (arrows point to the epicuticular wax crystals). Wax crystals were monitored at ×1000/× 25000 magnification (white boxes point to the magnified regions). **d** The expression of wax-synthesis-related genes in *MdMYB30* ectopic expression and WT Arabidopsis. **e** ChIP analysis with nuclei extracted from crosslinked 15-day-old apple calli of the empty vector (EV) and MYB30-GFP OE. The *Md18S* gene was used as a control, which provided the background level for the ChIP samples. P1, P2 and P3 represent putative binding MYB *cis*-elements in the *MdKCS1* promoter, respectively. Error bars represent standard deviation (SD; *n* = 3). Data are mean ± SD of three independent replicates. Different lowercase letters indicate significant differences at *P* < 0.05

Previous studies have demonstrated that *AtMYB30* is an important regulator of wax biosynthesis in Arabidopsis [20]. Total wax of *MdMYB30* ectopic expression and WT Arabidopsis was extracted to study the function of *MdMYB30* in wax accumulation. A significant difference was found in the total wax load between ectopically expressing *MdMYB30* and WT Arabidopsis. The total wax load was 1.5- to 1.3-fold higher in the stems and leaves of the *MdMYB30* OE lines compared to WT plants (Fig. 4a; Additional file 1: Figure S2a; Additional file 1: Table S1). Then, GC-MS was applied to determine the stem wax components. The analysis of cuticular wax composition showed that the contents of alkanes, alcohols, aldehydes, fatty acids, ketones, and esters increased in the *MdMYB30* ectopic expression lines compared with the WT. Among them, the increase in fatty acids was the largest, which was more than 2.6-fold higher (Fig. 4b; Additional file 1: Table S2). C29 alkanes, C31 alcohols, C29 aldehydes, C16 fatty acids, C29 ketones, and C29 and C30 esters increased significantly in the *MdMYB30* ectopic expression lines compared with those in the WT, providing evidence that *MdMYB30* plays an important role in cuticular wax biosynthesis (Additional file 1: Figure S2b).

We examined wax crystal morphology in the stems of *MYB30* ectopically expressing and WT plants, but did not find a difference in wax crystal morphology using SEM; however, the *MdMYB30* ectopically expressing Arabidopsis exhibited more epicuticular wax crystals than the WT (Fig. 4c). Similarly, the wax crystals in ectopically expressing Arabidopsis leaves were also examined, and just a small increase was detected (Additional file 1: Figure S2c). Therefore, expression of *MdMYB30* changed the numbers of wax crystals in stems and leaves.

Furthermore, the transcription levels of wax biosynthesis genes, including *AtHDG1*, *AtWRI1*, *AtCER3*, *AtWIN1*, *AtKCS1*, *AtACBP1*, *AtLACS2*, *AtCER1*, *AtSHINE2*, and *AtSHINE3* in the WT and *MdMYB30* ectopically expressed lines were detected by qRT-PCR. Ectopic expression of *MdMYB30* upregulated the transcription of several wax-related genes, such as *AtWRI1*, *AtWIN1*, *AtKCS1*, *AtACBP1*, *AtLACS2*, *AtSHINE2*, and *AtSHINE3*, indicating that *MdMYB30* promotes wax biosynthesis (Fig. 4d). Interestingly, *AtKCS1* was significantly upregulated in *MdMYB30* ectopic expression plants. Therefore, we analyzed the *MdKCS1* promoter sequence and found three motifs (P1, P2, and P3) that might interact with the MdMYB30 protein (Additional file 1: Figure S3). The ChIP-PCR assay was carried out to examine if MdMYB30 directly bound to the *MdKCS1* promoter. The results showed that MdMYB30 specifically bound to the TAATTT motif in the P3 sequence of the *MdKCS1* promoter to regulate wax synthesis (Fig. 4e).

### *MdMYB30* functions to change epicuticular wax properties and cuticular permeability

**Fig. 5:**
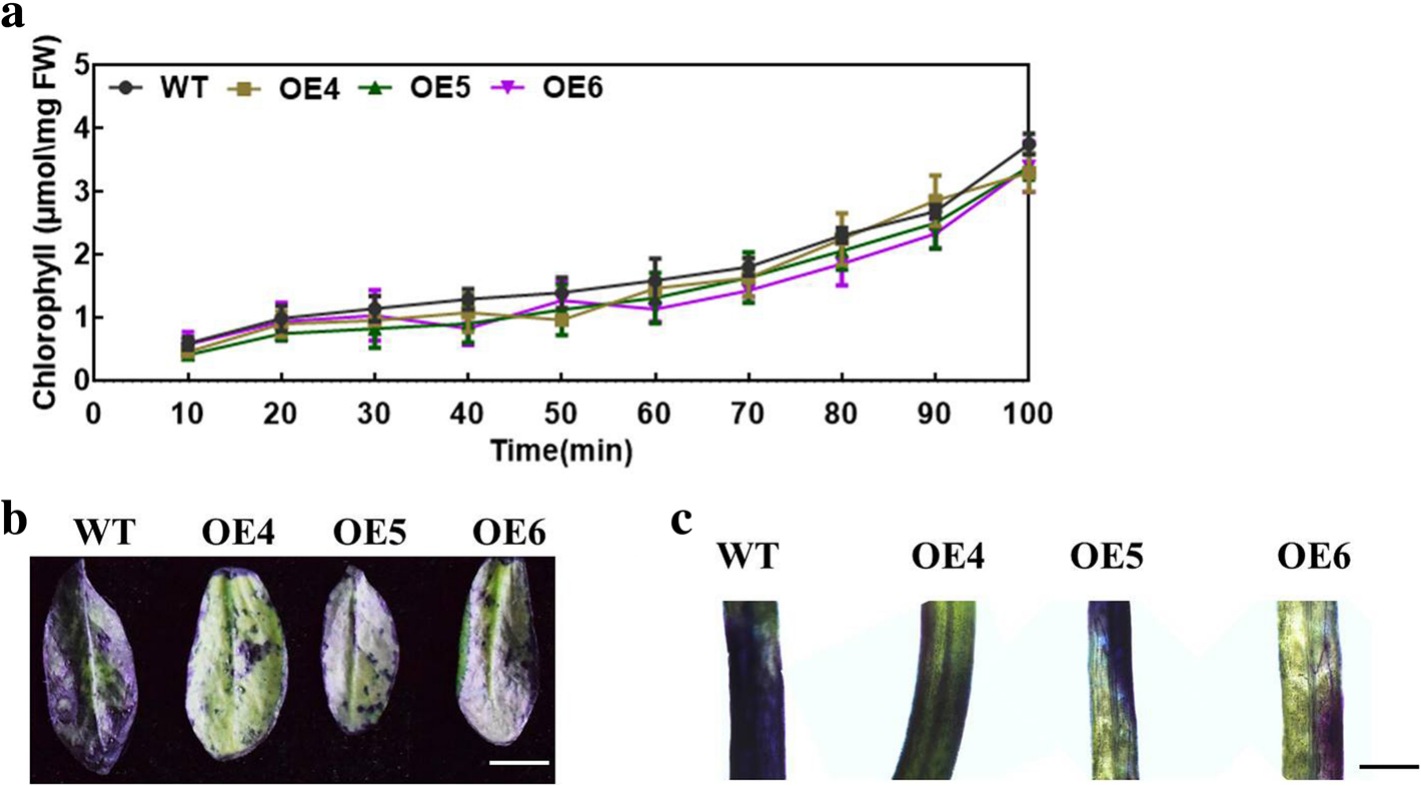
Leaf surface permeability of WT and *MdMYB30* ectopic expression plants. **a** Chlorophyll leaching rates in mature rosette leaves of WT and *MdMYB30* ectopic expression Arabidopsis chlorophyll μmol/mg FW (fresh weight = FW). **b** and **c** Toluidine blue staining of WT and *MdMYB30* ectopic expression Arabidopsis leaves **b** (scale bars: 1 cm) and stems **c** (scale bars: 500 μm)

The thickness of the wax layer is associated with cuticular permeability. The chlorophyll leaching assay was performed to investigate whether cuticular membrane properties in *MdMYB30* ectopically expressing plants changed. The results indicated that chlorophyll was extracted at a slower rate from *MdMYB30* ectopically expressing plants compared to WT (Fig. 5a), suggesting higher cuticular resistance for chlorophyll leaching in the ectopically expressing Arabidopsis. Subsequently, a toluidine blue staining experiment suggested that the *MdMYB30* ectopically expressing lines were more difficult to stain than the WT, regardless of whether rosette leaves or stems were stained (Fig. 5b, c). These results indicate that *MdMYB30* notably changed the permeability of the cuticular wax.

### *MdMYB30* confers resistance to *Pst* DC3000

**Fig. 6:**
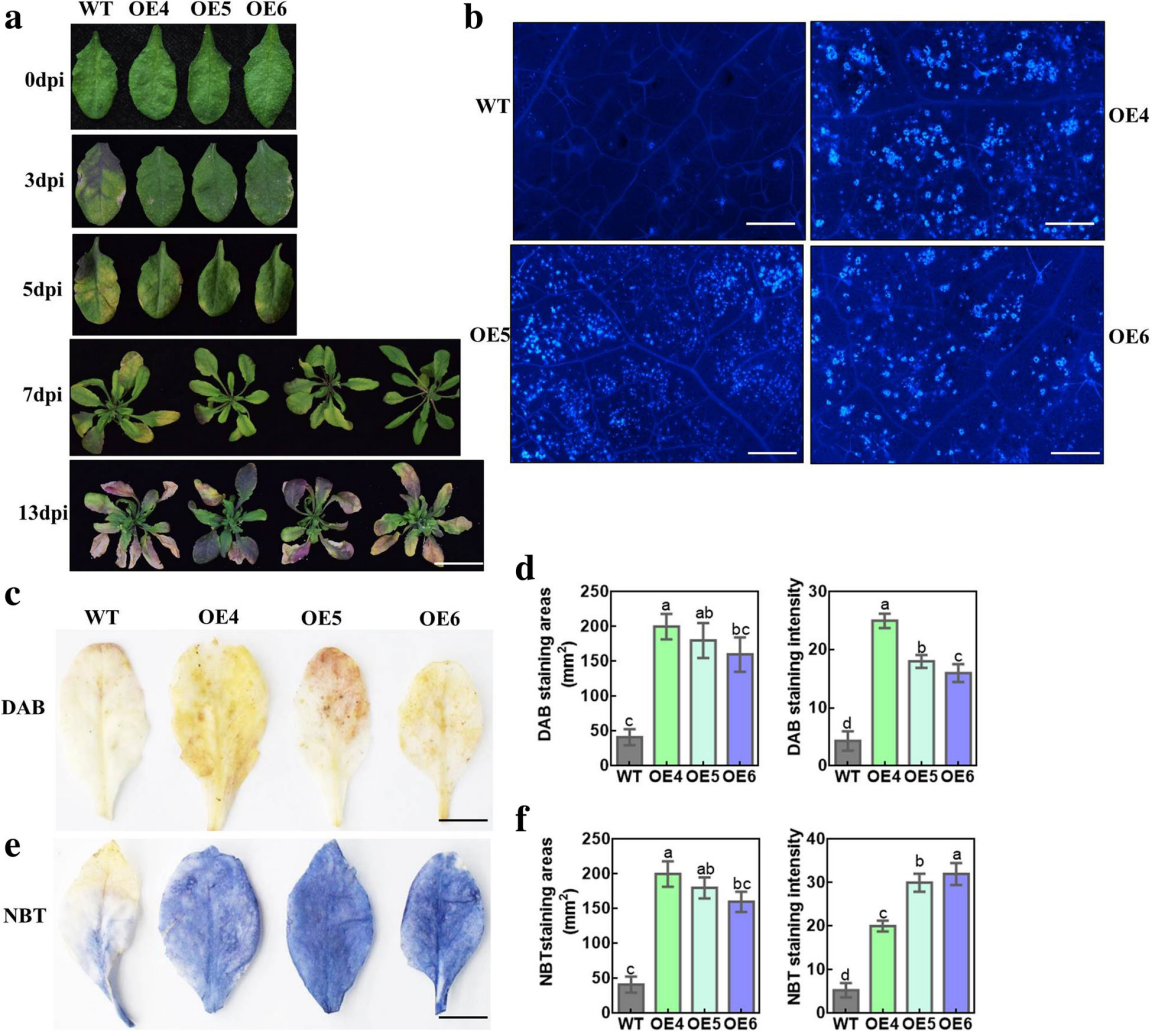
Ectopic expression of *MdMYB30* in Arabidopsis increases resistance to *Pst* DC3000. **a** Phenotypes of WT, *MdMYB30* OE4, OE5, and OE6 infected with *Pst* DC3000 for 0, 3, 5, 7, and 13 days (scale bars: 1 cm). **b** Callose deposition of WT and *MdMYB30* ectopic expression Arabidopsis infected with *Pst* DC3000 for 5 days (scale bars: 500 μm). **c** 3′,3′-Diaminobenzidine (DAB) staining for H_2_O_2_ in the leaves of WT and *MdMYB30* ectopic expression Arabidopsis infected with *Pst* DC3000 for 5 days. **d** DAB staining areas and intensity were determined with ImageJ software. **e** Nitroblue tetrazolium (NBT) staining for superoxide in the leaves of the WT and *MdMYB30* ectopic expression Arabidopsis infected with *Pst* DC3000 for 5 days. **f** NBT staining areas and intensity were determined with ImageJ software. Bars with different letters are significantly different at *P* < 0.05 according to Tukey’s single factor test. Data are mean ± standard deviation. At least three biological repetitions were performed

Cuticular wax acts as both a physical barrier against stressors and pathogens and is a chemical deterrent and activator of the plant defense response. We assessed whether *MdMYB30* affected resistance to *Pst* DC3000, which is a kind of bacterial pathogen. The results showed that WT leaves exhibited yellowing after 3 days of inoculation with *Pst* DC3000; however, the leaves of the *MdMYB30* ectopically expressing lines showed little yellowing. Susceptibility became more and more serious through time, and almost all WT rosette leaves developed chlorosis after 13 d of infection, whereas the leaves of the ectopically expressed lines were healthier at all time points examined, suggesting that *MdMYB30* ectopic expression conferred resistance to the pathogen (Fig. 6a). ROS levels are closely associated with a pathogen infection. To test whether the ectopically expressing lines had altered ROS levels after inoculation with *Pst* DC3000 for 5 d, the ROS reactive dyes DAB and NBT were used to test O_2_^−^ and H_2_O_2_ contents in infected rosette leaves. The results showed that ectopically expressing *MdMYB30* stained strongly, whereas weak staining was observed in the WT (Fig. 6c–f). Correspondingly, callose content improved in the ectopically expressing lines after the *Pst* DC3000 infection compared to the WT (Fig. 6b), demonstrating that *MdMYB30* enhanced resistance to *Pst* DC3000.

### *MdMYB30* positively regulates *B. dothidea* attack via transient transformation in apple fruits

**Fig. 7:**
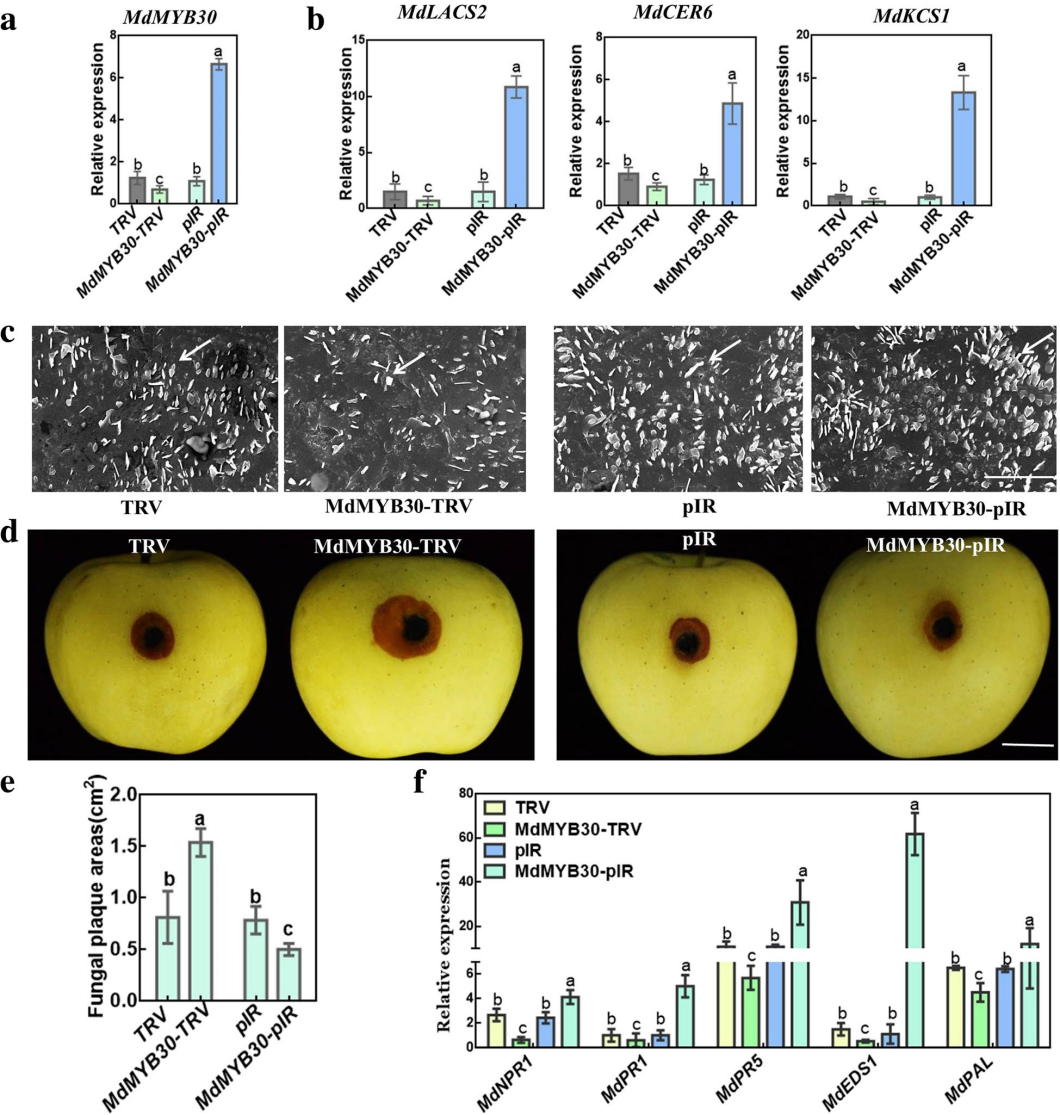
*MdMYB30* modulates cuticular wax-related resistance to *B. dothidea* in apple fruit. **a** Expression levels of *MdMYB30* around the injection sites according to qRT-PCR assays. MdMYB30-pIR was the *MdMYB30* transient overexpression vector, while the MdMYB30-TRV was the *MdMYB30* transient antisense vector. Empty pIR and TRV vectors were used as controls. **b** Expression of wax-synthesis-related genes around the injection sites. **c** Scanning electron microscopic images of cuticular wax crystal patterns of infected apple fruit peel around the injection sites (scale bars: 10 μm). The numbers of wax crystals around the MdMYB30-TRV/MdMYB30-pIR injection sites were less/more than the TRV/pIR empty vector counterparts (arrows point to the epicuticular wax crystals). **d** Phenotypes of TRV, MdMYB30-TRV, pIR, and MdMYB30-pIR infected with *B. dothidea* for 4 days after the injection. (scale bars: 1 cm) **e** Fungal plaque areas of apple after being infected with *B. dothidea* for 4 days. **f** Expression of apple disease resistance-related genes with the qRT-PCR assay around the injection sites after infection with *B. dothidea* for 4 days

**Fig. 8:**
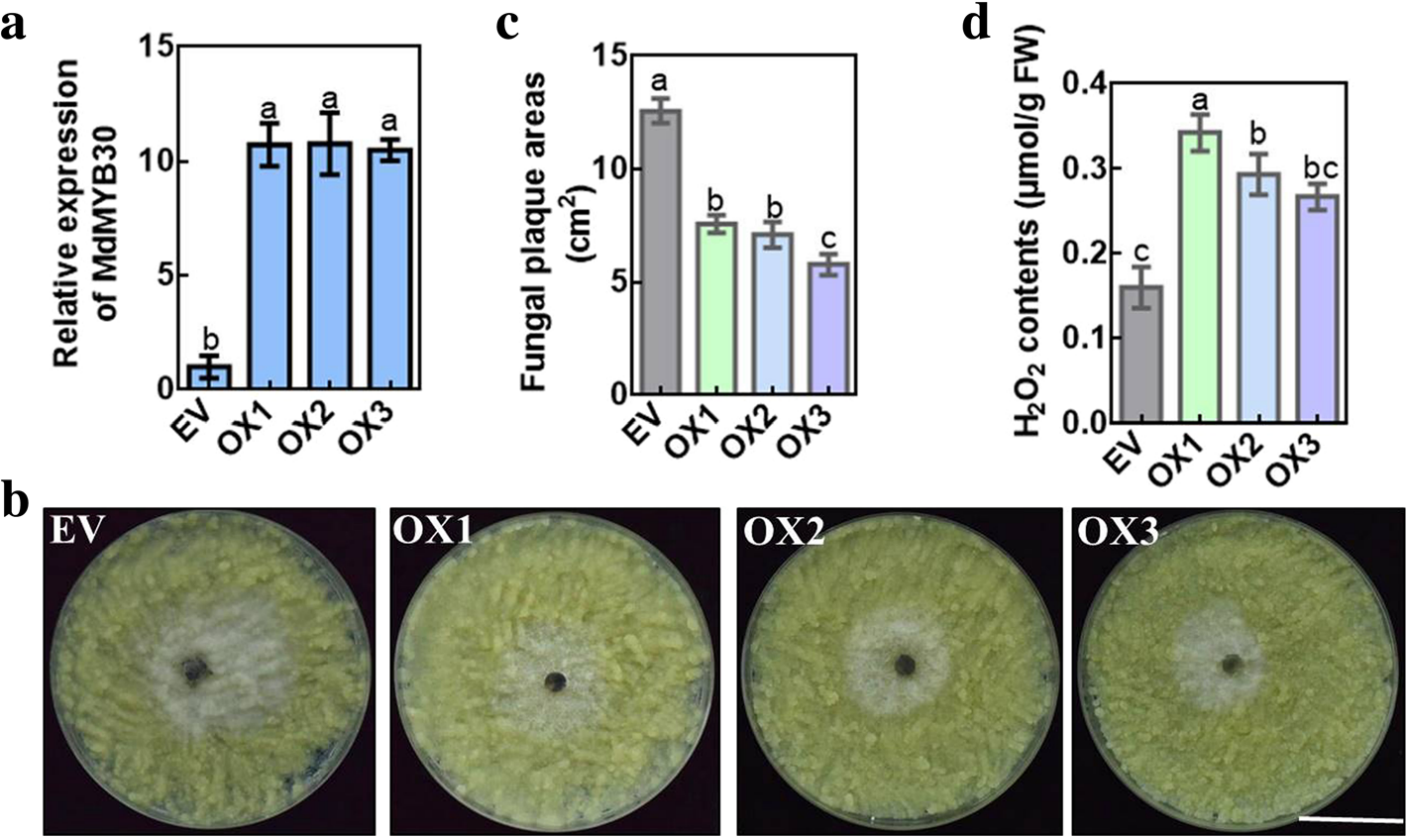
Overexpressing *MdMYB30* in apple calli increases resistance to *B. dothidea.* **a** Identification of transgenic apple calli expression level of *MdMYB30* by qRT-PCR. **b** Phenotypes of EV, *MdMYB30* OX1, OX2, and OX3 calli infected with *B. dothidea* for 4 days (scale bars: 1 cm). **c** Determination of fungal plaque areas of EV, *MdMYB30* OX1, OX2, and OX3 calli infected with *B. dothidea* for 4 days. **d** H_2_O_2_ contents of EV, *MdMYB30* OX1, OX2, and OX3 calli infected with *B. dothidea* for 4 days. Bars with different letters are significantly different at *P* < 0.05 according to Tukey’s single factor test. Data are mean ± standard deviation. At least three biological repetitions were performed

The expression of *MdMYB30* was increased or repressed using viral vector-based transient transformation to confirm the function of *MdMYB30* in enhancing resistance against pathogens in apple. Here, independent MdMYB30-TRV and MdMYB30-pIR transient expression of *MdMYB30* mRNA levels was downregulated or significantly upregulated, respectively (Fig. 7a). First, we detected the transcription of wax-related genes at the injection point, and three wax-related genes were positively correlated with *MdMYB30* expression (Fig. 7b). Furthermore, overexpression of *MdMYB30* accelerated the numbers of wax crystals around the injection sites according to SEM, compared to the control (Fig. 7c). Correspondingly, overexpression of *MdMYB30* decreased significantly, while its suppression increased the lesions, compared to the empty vector (Fig. 7d, e). The qRT-PCR results demonstrated that transcription of pathogenesis-related genes, including *MdNPR1*, *MdPR1*, *MdPR5*, *MdEDS1*, and *MdPAL* decreased at the TRV-MdMYB30 injection sites and increased at the pIR-MdMYB30 injection sites compared with the controls (Fig. 7f). Subsequently, the function of *MdMYB30* in disease resistance was confirmed in apple calli. We overexpressed *MdMYB30* in apple calli and obtained three *MdMYB30* overexpressing (*MdMYB30* OX) transgenic lines (Fig. 8a). Then, we tested the immunity of apple calli to *B. dothidea*, and found that overexpressing *MdMYB30* notably reduced the lesions compared to that of the empty vector (Fig. 8b, c). *MdMYB30* OX transgenic apple calli had higher levels of H_2_O_2_ than Empty Vector (Fig. 8d). These findings demonstrate that *MdMYB30* positively regulates wax accumulation in apple fruit, and strengthened apple resistance to *B. dothidea.*

## Discussion

Epicuticular wax participates in many physiological processes and is the main protective substance in the aerial parts of plants. MYB TFs participated in numerous processes [6, 21, 22]. Several R2R3 type MYB TFs play essential roles in biological stress through a possible cuticular-dependent pathway [23, 24], but the role of R2R3-MYB proteins in disease resistance is poorly understood in apple.

Protein subcellular localization is closely related to its function. It was reported that many MYB proteins are localized in the nucleus. In wheat (*Triticum aestivum* L.), *TaMYB1* is localized in the nucleus as indicated by an in vivo subcellular targeting experiment in onion epidermal cells [25]. In Arabidopsis, *MYB21* and *MYB24* are localized in the nucleus where they interact with *MYC2*/*3*/*4*/*5* proteins to modulate stamen growth and seed production through a bHLH-MYB complex [26]. The nuclear localized transcription factor MYB2 interacts with bHLH proteins in *Apium graveolens* to regulate anthocyanin biosynthesis [27]. In this study, the MdMYB30 fluorescence signal indicated that it was located in the nucleus, demonstrating that MdMYB30 as a transcription factor (TF) might target the genes localized in the nucleus that are involved in wax synthesis.

Plant MYB TFs play important roles in regulatory pathways and networks facing various stressors. In *L. purpureus*, *LpMYB1* ectopically expressing Arabidopsis exhibits enhanced drought and salt resistance compared to WT [28]. In Arabidopsis, AtMYC2 and/or AtMYB2 overexpressing plants show higher sensitivity to ABA and several ABA-inducible genes are upregulated, indicating that both proteins transcriptionally activate the expression of the ABA-inducible gene under drought stress [22]. In our study, ectopic expression of *MdMYB30* in Arabidopsis improved the responses to abiotic (PEG and ABA) and biotic (flg22 and *B. dothidea)* stressors.

Previous studies have shown that cuticular wax is mainly composed of alkanes, alcohols, aldehydes, free acids, ketones, and esters. Among them, alkanes account for 40–60%, alcohols account for 15–20%, aldehydes account for 5–10%, free acids account for 5–9%, ketones account for 15–20%, and esters account for < 10% of wax content. Increased alkane content in Arabidopsis has the greatest impact on total wax quality [29–31]. Our results show that wax content of each component is consistent with previous ones and the most obvious increase in wax content was fatty acids, compared to WT. We suspect that fatty acids may play a role in disease resistance of the plant epidermis. Studies of Arabidopsis long-chain acyl-CoA synthetase2 (*LACS2*) mutants have demonstrated that modifications in epidermal wax component, structure, and permeability may result in strong resistance to *Botrytis cinerea* [32]. An increase in wax content usually causes changes in the crystalline structure and quantity of wax crystals [33, 34]. Here, epidermal wax load and the number of wax crystals increased in *MdMYB30* ectopic expression Arabidopsis, which was possibly due to altered epidermal wax composition.

Previous reports indicate that the *AtKCS1*, *AtFDH*, and *AtDH3* genes are involved in VLCFA biosynthetic pathways. MYB30 directly activates the promoters of these genes to modulate cell death by increasing the biosynthesis of VLCFAs in the endoplasmic reticulum [5]. Transcription of the cuticular wax-related gene MYB96 was induced by drought and ABA and specifically bound to the promoter regions of several structural genes associated with wax biosynthesis*.* The increased wax content subsequently leads to enhanced drought tolerance in plants [35]. Our results demonstrate that enzyme-encoding genes and TFs increased significantly in *MdMYB30* ectopic expression Arabidopsis compared to WT. Among them, *AtKCS1* was the most obvious, which is consistent with *MdMYB30* binding to the *MdKCS1* promoter*,* thereby activating its expression.

As toluidine blue directly enters plant tissues without a solvent, this method can be used to study permeability of the cuticular membrane [32]. We used the toluidine blue staining assay to determine epidermal wax permeability. A previous study showed that *Vicia faba* leaves infected with bean yellow mosaic virus exhibit higher H_2_O_2_ and MDA levels compared to controls [36]. Here, the *MdMYB30* transgenic apple calli and ectopically expressing Arabidopsis leaves exhibited more O_2_^−^ and H_2_O_2_ contents than the WT after treatment with *Pst* DC3000 and *B. dothidea* for 4 days, respectively. Actually, a burst of ROS is considered one of the earliest responses to a biotic stress [37]. The ROS burst enhances plant disease resistance and increases changes in the cell walls [38]. These findings support previous views and confirm that *MdMYB30* positively regulates plant disease resistance.

## Conclusions

**Fig. 9:**
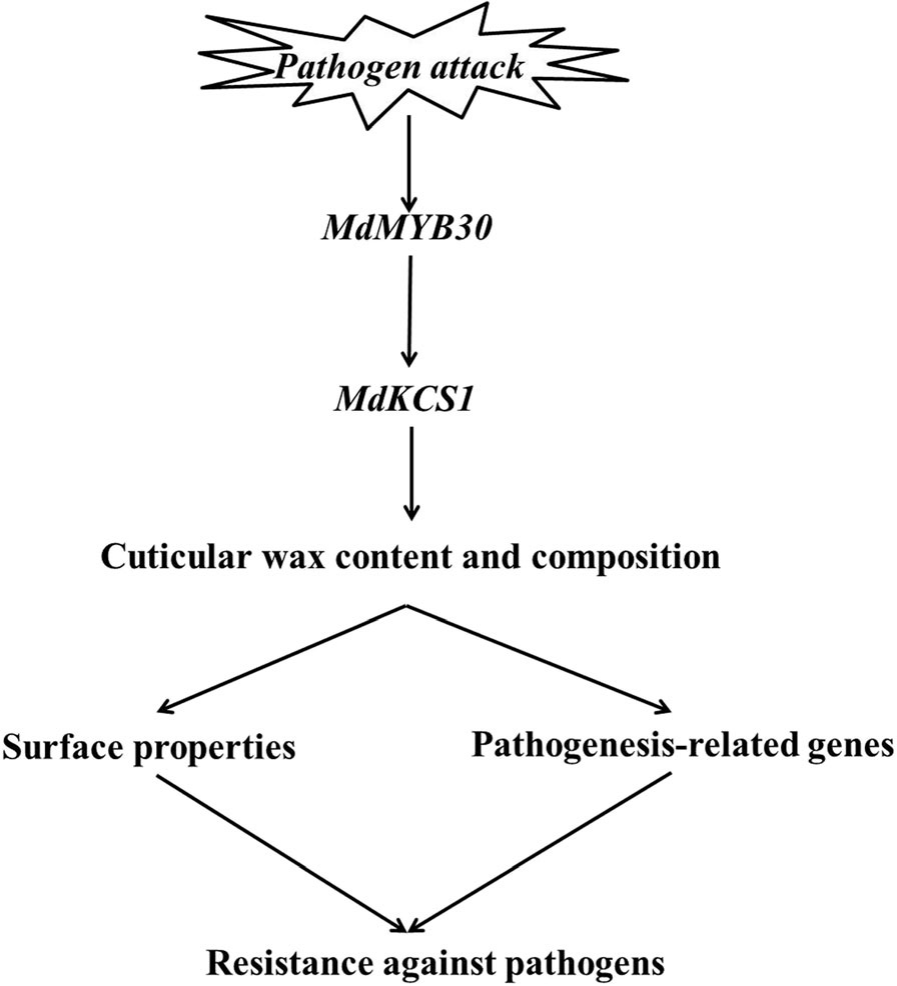
A model of *MdMYB30* regulated wax-related pathogen resistance

In conclusion, we present evidence that *MdMYB30* improved plant disease resistance and we propose a working model to explain this process (Fig. 9). Briefly, the TF *MdMYB30* regulates the biosynthesis of cuticular wax by activating the expression of *MdKCS1*. Thus, ectopically expressing *MdMYB30* significantly increased the total amount of cuticular wax and changed its composition; therefore, changing the surface properties and pathogenesis-related genes to resist against pathogens.

## Methods

### Plant materials and growth conditions

‘Orin’ apple calli (wild type) were cultured in the dark at 23–25 °C and subcultured every 3 weeks. The ‘Royal Gala’ tissue culture seedlings were cultured at 23–25 °C under a 16 h/8 h photoperiod and subcultured once per month. The subcultured mediums used were followed by [39]. Arabidopsis (ecotype ‘Columbia’) were grown in a long-day (16 h/8 h) light incubator at 19–22 °C. Tobacco (*Nicotiana benthamiana*) was grown at 23–25 °C under 14 h/10 h photoperiod.

Individual apple tissues were taken from 5-year-old ‘Royal Gala’ trees for the tissue expression experiments. The apple seedling treatment method for the stress response experiments followed that described previously [40]. The apple fruits were sampled 140 days after blooming from ‘Golden Delicious’ trees. *B. dothidea* was grown on potato dextrose agar (PDA) medium at 24 °C [41].

‘Orin’ apple calli was given by Prof. Takaya Moriguchi of the National Institute of Fruit Tree Science, Japan. ‘Royal Gala’ trees were obtained from the experimental station of Shandong Agricultural University. ‘Golden Delicious’ fruits were obtained from a commercial orchard near Tai-An City. We declare that the collection of plant materials comply with institutional, national, or international guidelines.

### Phylogenetic tree construction and amino-acid sequence analysis

The homologs of Arabidopsis MYB30 were obtained from the Protein BLAST program (http://www.ncbi.nlm.nih.gov/BLAST/). The phylogenetic tree and multiple sequence alignments were made using MEGA 7.0 and DNAMAN 6.0 software, respectively. MEME Suite (http://meme-suite.org/) software was used to obtain the functional motifs of the MYB30S. The MdMYB30 protein structural domain was predicted through NCBI website (https://www.ncbi.nlm.nih.gov/Structure/cdd/wrpsb.cgi).

### Plasmid construction and plant genetic transformation

Leaves of ‘Royal Gala’ tissue-cultured seedlings were used to extract RNA to clone MdMYB30 cDNA. The 1026 bp MdMYB30 cDNA was amplified using the MdMYB30-PRI-F (5′-GTCGACATGGGGAGGCCTCCTTGCT-3′) and MdMYB30-PRI-R (5′-GGATCC GAAAAAGTTAGCATTTCCATGATCT-3′) primers, designed with the SalI and BamHI restriction endonuclease sites, respectively. To obtain the MdMYB30-GFP fusion protein, the amplification product was inserted into PRI-GFP. Transformations of apple calli and Arabidopsis followed the methods described in [42, 43], respectively.

### Subcellular localization

The full length MdMYB30 sequence was inserted into the GFP vector to construct the recombinant plasmid MdMYB30-GFP. 35S::MdMYB30-GFP, which was used to transform tobacco [44]. The fluorescent signals were detected using a laser scanning confocal microscope (Zeiss LSM 510 META, Jena, Germany).

### Wax extraction, gas chromatography-mass spectrometry (GC-MS) analysis, and scanning electron microscopy (SEM)

Cuticular wax was extracted exhaustively using the following steps: chloroform extraction, nitrogen blow drying, derivatization reaction, and sample analysis following that described previously [39, 45]. SEM was performed as described previously [39]. The steps can be summarized as: sampling, vacuum freeze drying, gold spray treatment, observation of the mirror.

### Chromatin immunoprecipitation (ChIP)-polymerase chain reaction (PCR) analysis

The ChIP-PCR analysis was performed using the EpiTect ChIP OneDay Kit following the method described by [46]. The primers used in the ChIP-PCR analysis are listed in Additional file 1: Table S3.

### Gene expression analysis

Plant total RNAs were isolated using the RNA plant Plus Reagent Kit (Tiangen, Beijing, China) and TRIzol reagent (Invitrogen, Carlsbad, CA, USA). Reverse transcription was performed using the PrimeScript™ RT reagent Kit with the gDNA Eraser (TaKaRa, Shiga, Japan). The quantitative RT-PCR assay was carried out using the Step One Plus™ Real-Time PCR System (Thermo Fisher Scientific, Waltham, MA, USA) to examine the transcription level of the wax and disease resistance-related genes. The qRT-PCR was performed according to [47]. The primers used for qRT-PCR are listed in Additional file 1: Table S3.

### Chlorophyll leaching assay

The rosettes from 4-week-old seedlings were sampled. Chlorophyll content was determined following methods described previously [48, 49].

### Toluidine blue staining

Toluidine blue staining was performed to detect leaf surface permeability, and carried out according to [45].

### Pathogen infection assays

Leaves of WT and transgenic Arabidopsis were sampled and infected as described previously [50]. The Arabidopsis infection analysis with the bacterial strain *Pseudomonas syringae* pv tomato DC3000 (*Pst* DC3000) was carried out as described by [51]. *Pst* DC3000 is a model bacterial strain for the interaction between Arabidopsis and pathogenic bacteria. The inoculation of apple calli with *B. dothidea* was performed according to [50].

### ROS assays

H_2_O_2_ was quantified according to the method described by [52]. Nitroblue tetrazolium (NBT) and 3,3-diaminobenzidine (DAB) staining methods were applied to determine O_2_^−^and H_2_O_2_ levels, respectively [50, 53].

### Callose staining

Four days after infecting with *Pst* DC3000, the leaves were rinsed 20 h in fixative solution (formaldehyde: acetic acid: ethanol: H_2_O, 3.7%: 5%: 50%: 41.3%, by vol.). The sample was removed from the fixative and soaked with 8 M NaOH for 5–6 h; the NaOH was removed and the sample was washed with deionized water for 10 min; the water was removed, and 0.01% (w/v) aniline blue dye solution was added in the dark for 1 h. The quantitative statistics of the callose deposits was carried out using ImageJ software as described previously [50].

### Construction of the viral vectors and agroinfiltration of apple fruit

Viral vectors were used as described by [54]. The primer pairs MdMYB30-F (5′-ATGGGGAGGCCTCCTTGCT-3′)/−R (5′- GAAAAAGTTAGCATTTCCATGATCT-3′) were used to amplify the full-length MdMYB30, which was inserted into the pIR vector to generate the pIR-MdMYB30 construct (Additional file 1: Table S3). Then, a portion of the full-length cDNA of *MdMYB30* (15–348 bp) was cloned and inserted into the tobacco rattle virus (TRV) vector in an antisense orientation to obtain the antisense construct TRV-MdMYB30. The instant injection method was used as described by [55]. After the injection, the fruit was placed in the dark for 3 days at room temperature, after which *Botryosphaeria dothidea* was inoculated at the injection point with an inoculating loop, placed in the dark at room temperature under humid conditions, and the proliferation of *B. dothidea* was observed after 3 days.

### Statistical analysis

Each experiment was set up with three times repetitions and the data are based on the results of three parallel experiments. The data were analyzed for significance using Data Processing System (DPS) (http://www.chinadps.net/). Tukey’s single factor test was used. All datasets were analyzed in the same way. A *p*-value < 0.05 was considered significant.

## Additional file

Additional file 1: **Figure S1.** Schematic diagram of MdMYB30 genomic and cDNA sequences. UTR: untranslated region. **Figure S2.** Changes in wax load, composition, and cuticular wax crystal morphology of WT plants and *MdMYB30* ectopic expression plants detected by scanning electron microscopy. **a** Total cuticular wax content of leaves, calculated per unit area of 6-week-old Arabidopsis from the WT and *MdMYB30* ectopic expression lines. **b** Cuticular wax composition of alkanes, alcohols, aldehydes, fatty acids, ketones, and esters on the stem surfaces of WT and *MdMYB30* ectopic expression plants analyzed by GC-MS. Wax constituents are grouped by carbon chain length and chemical class. **c** Wax crystal morphology of 6-week-old Arabidopsis leaves from the WT and *MdMYB30* ectopic expression lines (scale bars: 10 μm). Error bars represent standard deviation (SD; *n* = 3). Data are mean ± SD of three independent replicates. Different lowercase letters indicate significant differences at *P* < 0.05. **Figure S3.** Diagram representing the genomic structure and primer sets (indicated by P1-P3) analyzed in the *MdKCS1* genes by ChIP-qPCR. White boxes represent primer sets, and black boxes represent ATG starting open reading frame (ORF). **Table S1.** Total epicuticular wax on wild type Arabidopsis, *MdMYB30* OE4, OE5, and OE6 surface areas. **Table S2.** Epicuticular wax component (μg/dm^2^) in stems of wild-type Arabidopsis, *MdMYB30* OE4, OE5, and OE6. **Table S3.** Primers used in this study. **File S1.** The amino acid sequences of MdMYB30 and homologs from 12 other plant species to analyze the phylogenetic relationships. (ZIP 1525 kb)

## Data Availability

All the data about the present study has been included in the table and/or figure form in the current manuscript or the supplement already. Authors are pleased to share analyzed/raw data and plant materials upon reasonable request.

## Abbreviations

*B. dothidea*: *Botryosphaeria dothidea*
ORF: Open reading frame
*Pst* DC3000: Bacterial strain *Pseudomonas syringae* pv *tomato*
ROS: Reactive oxygen species
SEM: Scanning electron microscopy
VLCFAs: Very-long-chain fatty acids
