# Supplementary material for: The R2R3 MYB transcription factor MdMYB30 modulates plant resistance against pathogens by regulating cuticular wax biosynthesis
Source: BMC Plant Biol. 2019 Aug 19;19:362. doi: 10.1186/s12870-019-1918-4 (PMC6700842; doi:10.1186/s12870-019-1918-4)
Supplement: Supplementary file 1 — Figure S1. Schematic diagram of MdMYB30 genomic and cDNA sequences. UTR: untranslated region. Figure S2. Changes in wax load, composition, and cuticular wax crystal morphology of WT plants and MdMYB30 ectopic expression plants detected by scanning electron microscopy. a Total cuticular wax content of leaves, calculated per unit area of 6-week-old Arabidopsis from the WT and MdMYB30 ectopic expression lines. b Cuticular wax composition of alkanes, alcohols, aldehydes, fatty acids, ketones, and esters on the stem surfaces of WT and MdMYB30 ectopic expression plants analyzed by GC-MS. Wax constituents are grouped by carbon chain length and chemical class. c Wax crystal morphology of 6-week-old Arabidopsis leaves from the WT and MdMYB30 ectopic expression lines (scale bars: 10 μm). Error bars represent standard deviation (SD; n = 3). Data are mean ± SD of three independent replicates. Different lowercase letters indicate significant differences at P < 0.05. Figure S3. Diagram representing the genomic structure and primer sets (indicated by P1-P3) analyzed in the MdKCS1 genes by ChIP-qPCR. White boxes represent primer sets, and black boxes represent ATG starting open reading frame (ORF). Table S1. Total epicuticular wax on wild type Arabidopsis, MdMYB30 OE4, OE5, and OE6 surface areas. Table S2. Epicuticular wax component (μg/dm2) in stems of wild-type Arabidopsis, MdMYB30 OE4, OE5, and OE6. Table S3. Primers used in this study. File S1. The amino acid sequences of MdMYB30 and homologs from 12 other plant species to analyze the phylogenetic relationships. (ZIP 1525 kb) [file 12870_2019_1918_MOESM1_ESM.zip › Additional file 1 Table S1..docx]

**Supplementary Table S1** Total epicuticular wax on wild type Arabidopsis, *MdMYB30* OE4, OE5, and OE6 surface areas. Values represent epicuticular wax load in μg/dm^2^ of total leaf and stem. Error bars represent standard deviation (SD; *n* = 3). Data are the mean ± SD of three independent replicates. Lowercase letters indicate significant differences at *P* < 0.05

|  | WT | OE4 OE5 | OE6 |
| --- | --- | --- | --- |
|  |  |  |  |
| stem  leaf | 1008.0000^b^  ±140.0817  134.6670^b^  ±10.2051 | 1399.3330^a^ 1402.0000^a^  ±130.4109 ±140.5715      181.000^a^ 172.3330^a^  ±20.0817 ±20.2495 | 1492.0000^a^  ±150.496  179.6670^a^  ±10.1700 |
|  |  |  |  |
